# Inflammation and Cancer: From the Development of Personalized Indicators to Novel Therapeutic Strategies

**DOI:** 10.3389/fphar.2022.838079

**Published:** 2022-03-03

**Authors:** Patrizia Ballerini, Annalisa Contursi, Annalisa Bruno, Matteo Mucci, Stefania Tacconelli, Paola Patrignani

**Affiliations:** ^1^ Center for Advanced Studies and Technology (CAST), Chieti, Italy; ^2^ Department of Innovative Technologies in Medicine and Dentistry, Chieti, Italy; ^3^ Department of Neuroscience, Imaging and Clinical Science, G. d’Annunzio University, Chieti, Italy

**Keywords:** colorectal cancer, HCC, aspirin, COX-2, platelets, inflammation, NSAIDs, drug delivery

## Abstract

Colorectal (CRC) and hepatocellular carcinoma (HCC) are associated with chronic inflammation, which plays a role in tumor development and malignant progression. An unmet medical need in these settings is the availability of sensitive and specific noninvasive biomarkers. Their use will allow surveillance of high-risk populations, early detection, and monitoring of disease progression. Moreover, the characterization of specific fingerprints of patients with nonalcoholic fatty liver disease (NAFLD) without or with nonalcoholic steatohepatitis (NASH) at the early stages of liver fibrosis is necessary. Some lines of evidence show the contribution of platelets to intestinal and liver inflammation. Thus, low-dose Aspirin, an antiplatelet agent, reduces CRC and liver cancer incidence and mortality. Aspirin also produces antifibrotic effects in NAFLD. Activated platelets can trigger chronic inflammation and tissue fibrosis via the release of soluble mediators, such as thromboxane (TX) A_2_ and tumor growth factor (TGF)-β, and vesicles containing genetic material (including microRNA). These platelet-derived products contribute to cyclooxygenase (COX)-2 expression and prostaglandin (PG)E_2_ biosynthesis by tumor microenvironment cells, such as immune and endothelial cells and fibroblasts, alongside cancer cells. Enhanced COX-2-dependent PGE_2_ plays a crucial role in chronic inflammation and promotes tumor progression, angiogenesis, and metastasis. Antiplatelet agents can indirectly prevent the induction of COX-2 in target cells by inhibiting platelet activation. Differently, selective COX-2 inhibitors (coxibs) block the activity of COX-2 expressed in the tumor microenvironment and cancer cells. However, coxib chemopreventive effects are hampered by the interference with cardiovascular homeostasis via the coincident inhibition of vascular COX-2-dependent prostacyclin biosynthesis, resulting in enhanced risk of atherothrombosis. A strategy to improve anti-inflammatory agents’ use in cancer prevention could be to develop tissue-specific drug delivery systems. Platelet ability to interact with tumor cells and transfer their molecular cargo can be employed to design platelet-mediated drug delivery systems to enhance the efficacy and reduce toxicity associated with anti-inflammatory agents in these settings. Another peculiarity of platelets is their capability to uptake proteins and transcripts from the circulation. Thus, cancer patient platelets show specific proteomic and transcriptomic expression profiles that could be used as biomarkers for early cancer detection and disease monitoring.

## Introduction

Inflammation is a physiological protective response to various harmful stimuli, such as pathogens, damaged cells, and toxic compounds, that involves innate and adaptive immune systems. Acute inflammation is a self-limiting process that can develop into chronic inflammation, whether it is persistently unresolved (Furman et al., 2019). Chronic inflammation promotes many pathological conditions, including atherothrombosis (Libby et al., 2009; Ridker 2019), tissue fibrosis (Wynn and Ramalingam 2012), aberrant angiogenesis (Jackson et al., 1997), and neoplasia (Coussens and Werb, 2002; Grivennikov et al., 2010; Hanahan and Weinberg 2011).

Gawatz et al. (2005) have highlighted the molecular machinery and inflammatory pathways that platelets use to initiate and accelerate atherothrombosis. The pioneering intuition of Rudolf Virchow, who, in 1863, formulated the hypothesis of a link between microinflammation and subsequent cancer development, has been confirmed by numerous clinical and experimental data (Coussens and Werb, 2002; Grivennikov et al., 2010; Hanahan and Weinberg, 2011). Recently, the role of platelets in driving chronic intestinal inflammation and fibrosis has been elegantly demonstrated by Sacco et al. (2019) by generating a mouse with the specific deletion of cyclooxygenase (COX)-1 in megakaryocytes/platelets (platelet COX-1 cKO mouse) treated with dextran sodium sulfate (DSS) to induced colitis. The platelet COX-1 cKO mouse has a phenotype resembling that induced by the antiplatelet agent low-dose Aspirin in humans. The drug causes complete and persistent inhibition of platelet COX-1 with a limited inhibitory effect on vascular COX-2-dependent prostacyclin [prostaglandin (PG)I_2_] (Patrignani et al., 1982; Capone et al., 2004).

**FIGURE 1:**
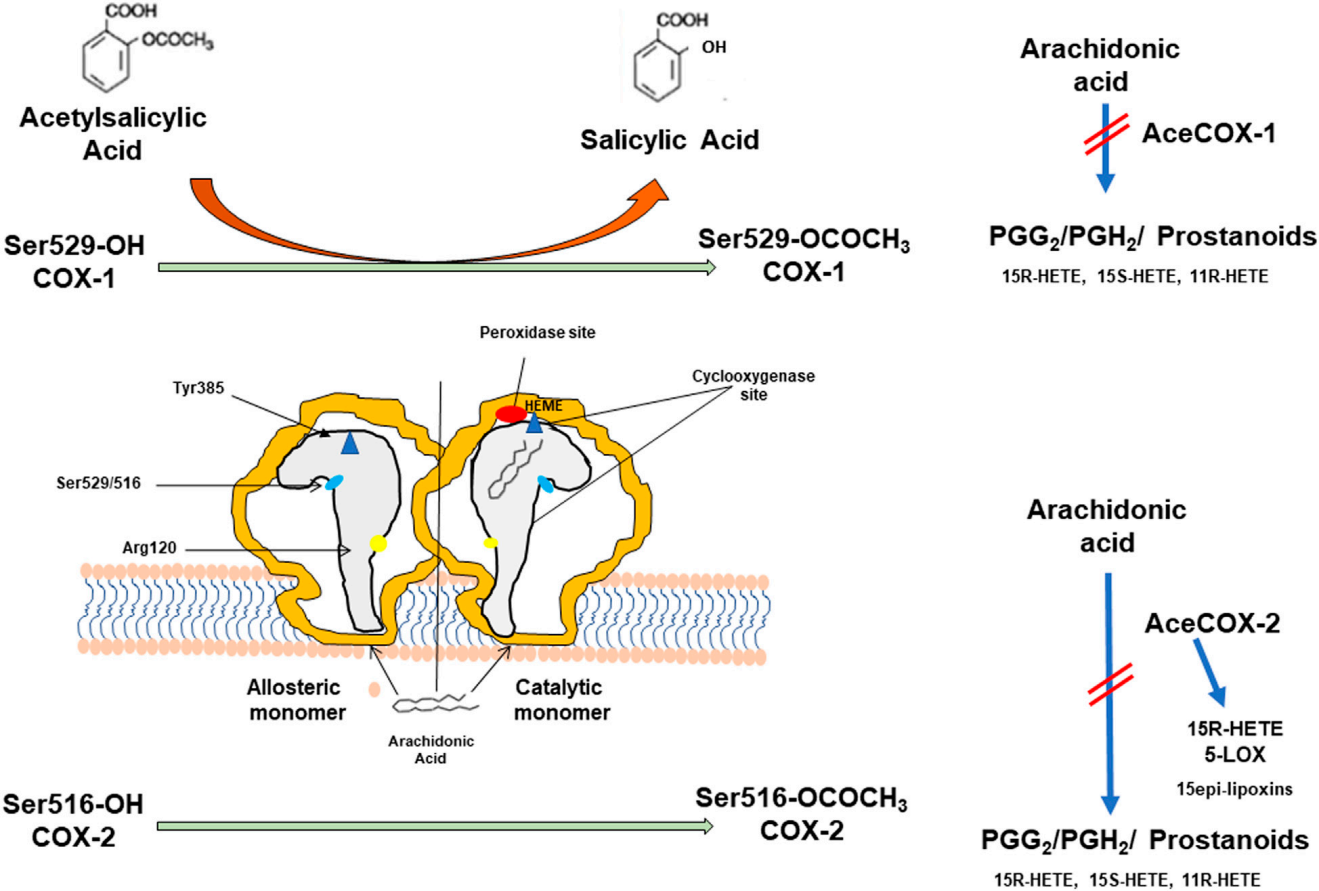
Aspirin (acetylsalicylic acid, ASA) acts by irreversibly acetylating cyclooxygenase (COX)-1 and COX-2. Prostaglandin H synthase (PGHS)-1 and -2 (also known as COX-1 and COX-2) are microsomal homodimeric heme glycoproteins that catalyze two key reactions in the biosynthesis of prostanoids: the bis-dioxygenation of arachidonic acid to form PGG_2_ (by the cyclooxygenase activity) and the reduction of PGG_2_ to PGH_2_ (by the peroxidase activity). In addition to PGH_2_, the monohydroxy acids 11(R)-hydroxyeicosatetraenoic acid 11(R)-hydroxyeicosatetraenoic acid 11R-(HETE), 15S-HETE, and 15R-HETE are generated as minor products of the cyclooxygenase reaction. COX-isozymes function as conformational heterodimers comprised of a regulatory allosteric monomer and a catalytic monomer. The catalytic monomer has a bound heme, whereas the allosteric monomer does not. The cyclooxygenase active site is a hydrophobic channel (reported in grey), and Arg-120 is located near its mouth. Arg-120 forms an ionic bond with the carboxylate group of arachidonate, and this interaction is an important contributor to the overall strength of arachidonate binding to COX-1 more than COX-2. Arg-120 is also the binding site for Aspirin. At the top of the channel is located Tyr-385, which is the critical catalytic amino acid for the cyclooxygenase reaction. Ser-529 and Ser-516 are the aminoacids acetylated by Aspirin. The acetylation of COX-isozymes is associated with the formation of salicylic acid, a weak COX inhibitor. The acetylation of only one monomer of COX dimer is sufficient for causing profound inhibition of cyclooxygenase activity. The acetylation of COX-1 at Ser-516 inhibits the catalytic activity of cyclooxygenase and prevents the generation of PGG_2_. In contrast, acetylated COX-2 at Ser-516 gains a novel catalytic activity and forms 15 R-HETE. 15R-HETE could be transformed to 15(R)epi-lipoxin (LX)A_4_ and 15epi-LXB_4_ in cells expressing the 5-lipoxygenase (5-LOX), the enzyme also responsible for leukotriene biosynthesis.

Aspirin inhibits platelet function by causing an irreversible inactivation of COX-1 through the acetylation of a serine residue at position 529 of the COX active site (Figure 1). Once acetylated, COX-1 is inhibited for the lifetime of the anucleate platelets since they have a limited capacity to generate new protein. Thus, low-dose Aspirin causes a virtually complete inhibition of platelet thromboxane (TX) A_2_ biosynthesis, a primary agonist of platelet aggregation, throughout dosing intervals (24 h), even if the drug has a short half-life (Pedersen and FitzGerald 1984). Aspirin also acetylates COX-2 at serine 516, and the enzyme gains a new catalytic activity with the formation of 15R-hydroxyeicosatetraenoic acid (HETE) from arachidonic acid (AA) (Lecomte et al., 1994) (Figure 1). However, we have recently shown that in the human colon cancer cell line, HCA-7, stimulated with 0.5 μM of AA *in vitro*, Aspirin causes concentration-dependent acetylation of COX-2 at serine 516 and inhibition of PGE_2_ production with comparable EC_50_ values (concentration causing 50% of maximal effect: 19.84 and 19.58 μM, respectively) while 15R-HETE is undetectable (Tacconelli et al., 2020). PGE_2_ plays a crucial role in chronic inflammation and promotes tumor progression, angiogenesis, and metastasis (Wang and Dubois 2018). Aspirin affects COX-2-dependent PGE_2_ with comparable potency to platelet COX-1 *in vitro* (Patrignani et al., 2017). However, when administered at low doses once a day, the drug targets mainly platelet COX-1 due to rapid *de novo* synthesis of COX-2 in nucleated cells (Patrignani et al., 2017). The development of direct biomarkers of aspirin action (i.e., the evaluation of the extent of acetylation of COX-1 at serine 529) (Li et al., 2014; Patrignani et al., 2014) has confirmed that low-dose Aspirin acts through a major effect on the platelet with a minor direct impact on the colorectal mucosa (Patrignani et al., 2017). Aspirin has been reported to cause anticancer effects by affecting different COX-independent molecular pathways (Alfonso et al., 2014; Ricciotti et al., 2021). However, these effects have been mainly found *in vitro* at supratherapeutic concentrations (millimolar concentrations).

The analysis of randomized clinical trials (RCTs) with Aspirin for cardiovascular (CV) prevention has shown that low doses are associated with a maximal efficacy to reduce incidence and mortality from colorectal cancer (CRC) over long-term follow-up (reviewed in Patrignani and Patrono 2016; Patrignani and Patrono 2018). This is like that found for Aspirin’s secondary prevention of atherothrombotic vascular events (Patrono et al., 2017). Thus, the pharmacodynamics features of low-dose Aspirin and the clinical data suggest that the inhibition of COX-1-dependent platelet function is a central mechanism for CV and intestinal tumorigenesis prevention (Patrignani and Patrono 2016; Patrignani and Patrono 2018).

Platelets can contribute to tumorigenesis and metastasis via the release of many soluble factors (including prostanoids, cytokines, growth, and angiogenic proteins and transcripts) and extracellular vesicles (EVs), which promote the change of the phenotype of stromal (such as fibroblasts and immune cells), endothelial, epithelial cells and cancer cells (Gay and Felding-Habermann 2011; Burnouf et al., 2014; Contursi et al., 2017; Dovizio et al., 2018). We have shown that platelets induce COX-2 expression and enhance PGE_2_ biosynthesis in colon cancer cells *in vitro* associated with epithelial-mesenchymal transition (EMT), increased migration, and metastatic potential when injected in mice (Dovizio et al., 2013; Guillem-Llobat et al., 2016). The overexpression of COX-2 is an important event for the development and progression of CRC (Wang and Dubois 2018). Importantly, COX-2-dependent PGE_2_ causes immunosuppression and tumor immune escape (Zelenay et al., 2015; Wang and Dubois 2018). Low-dose Aspirin cannot directly inhibit COX-2 activity but can restrain COX-2 induction in stromal cells and intestinal epithelial cells by inhibiting platelet activation (Patrignani and Patrono 2016; Dovizio et al., 2017; Patrignani and Patrono 2018).

Selective COX-2 inhibitors (coxibs) have antitumor effects (reviewed by Patrignani and Patrono, 2016), and they might be effective adjuvants for immune-based therapies in cancer patients (Zelenay et al., 2015; Wang and Dubois 2018). However, the chronic use of coxibs interferes with CV homeostasis due to the inhibition of vascular COX-2-dependent PGI_2_ biosynthesis and the resulting increased risk of atherothrombosis (Grosser et al., 2006). Platelet ability to transfer their contents to cancer cells might be used to develop platelet-based drug delivery systems (Lu et al., 2019), thus improving the efficacy of anti-inflammatory drugs and reducing their toxicity.

A peculiarity of platelets is that they can uptake many soluble components (proteins, transcripts, including microRNAs) from plasma, and this phenotypically distinct population is termed tumor-educated blood platelets (TEPs). TEPs originate as a systemic and local response to tumor growth, and their assessment has diagnostic potential (Roweth and Battinelli, 2021), indicating the individual’s clinical condition.

CRC and primary liver cancer are leading causes of cancer-related deaths worldwide (Bray et al., 2018). Therefore, their prevention and appropriate treatments represent an unmet medical need and a global health challenge. This review aims to put together evidence on the possible role of platelets in the development and perpetuation of chronic inflammation associated with CRC and hepatocellular carcinoma (HCC) and opening the way to therapeutic strategies involving antiplatelet agents and platelet-mediated drug delivery systems to enhance the efficacy and reduce the toxicity of anti-inflammatory agents. Finally, the evidence on the possible use of proteomics and transcriptomics expression profiles of TEPs as biomarkers for early cancer detection and disease monitoring is discussed.

## Colorectal Cancer

### Inflammation, Platelets, and CRC

CRC ranks third among the most diagnosed cancers accounting for 10% of the cancer burden (Bray et al., 2018). Two chronic relapsing inflammatory disorders of the gastrointestinal tract, Crohn’s disease (CD) and ulcerative colitis (UC), which represent the main subtypes of inflammatory bowel diseases (IBD), are associated with CRC development. For these patients, the relative risk for CRC is about two to three times greater than that of the general population (Carter et al., 2004) and correlates with the disease duration, increasing by 0.5–1% yearly, 8–10 years after diagnosis (Herszenyi et al., 2007). This indicates that chronic intestinal inflammation contributes to tumor formation. Indeed, differently from the wound healing process that resolves following proper immune cell recruitment and epithelial cell proliferation, growing tumors enter into a feed-forward loop of inflammation-induced signaling and inflammatory cell recruitment (Dovizio et al., 2017).

**FIGURE 2:**
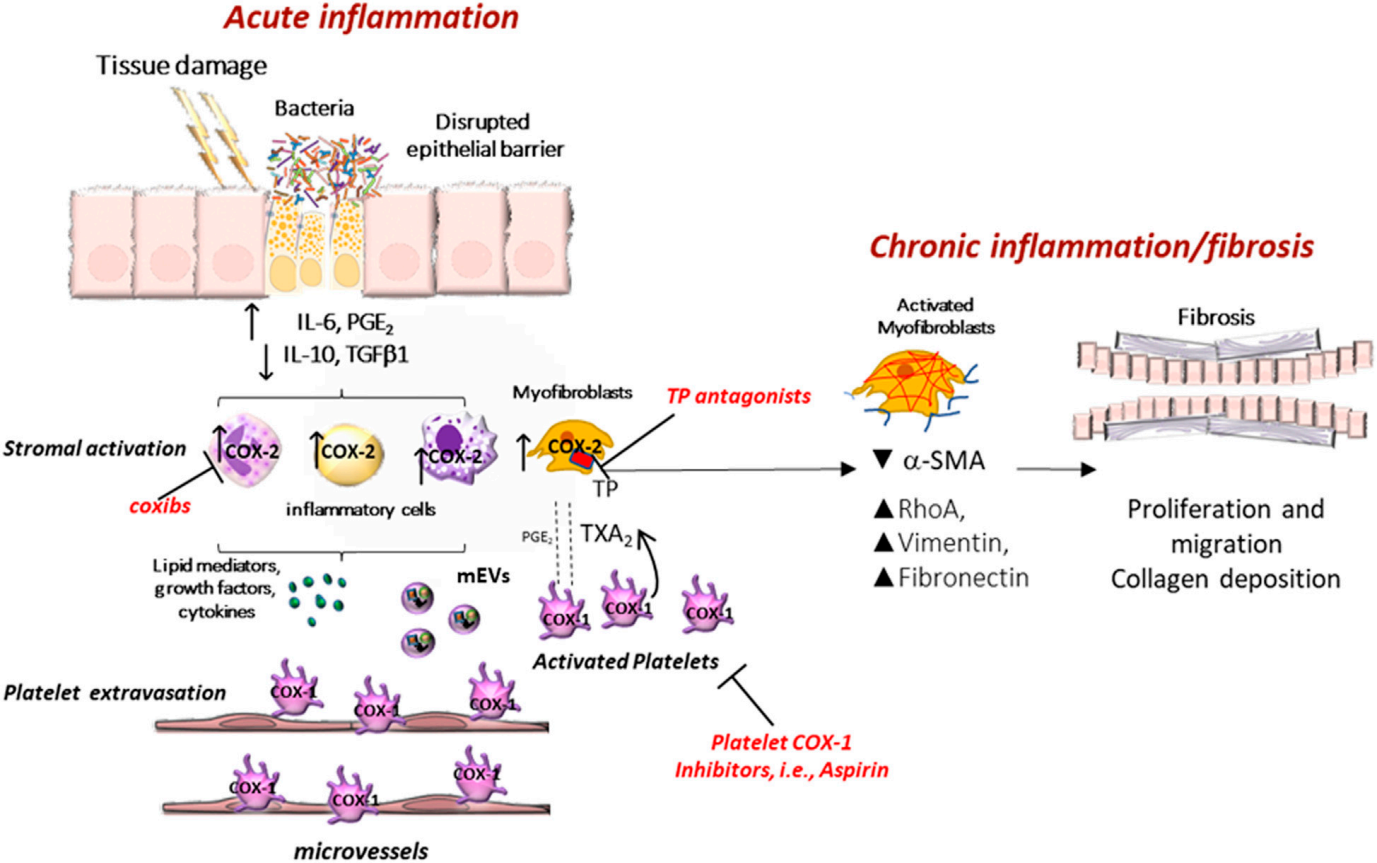
Clinical implications of eicosanoid inhibition in intestinal tumorigenesis. Activated platelets release soluble mediators, including thromboxane A_2_ (TXA_2_), prostaglandin E_2_ (PGE_2_), 12S-hydroxyeicosatetraenoic acid (12S-HETE), growth factors, cytokines, and platelet-derived medium-sized extracellular vesicles (mEVs) containing microRNAs, which contribute to the phenotypic changes of the cells in the stromal microenvironment. Interactions between epithelial cells and stromal cells undergo an alteration that induces the development of intestinal neoplasia. A crucial event is the enhanced biosynthesis of PGE_2_ in the intestinal mucosa, occurring in the early stages of tumor development via the cyclooxygenase (COX)-1 activity, in association with the suppression of the prostaglandin-degrading enzyme 15-prostaglandin dehydrogenase (15-PGDH). Later, the overexpression of COX-2 contributes to the generation of aberrant levels of PGE_2_ in the stromal compartment and epithelial cells. PGE_2_ plays multifaceted roles in cancer promotion, including proliferation, migration, epithelial-mesenchymal transition (EMT), and immune escape. Tumor cells that undergo this phenomenon acquire a migratory capacity that facilitates metastatic colonization. By inhibiting platelet activation, antiplatelet agents can indirectly restrain the induction of COX-2 in target cells. Differently, selective COX-2 inhibitors (coxibs) affect COX-2 activity already expressed in the tumor microenvironment and cancer cells. 12S-HETE generated by 12-lipoxygenase (LOX) may have protumorigenic effects, and selective 12-LOX inhibitors are in clinical development.

Platelets can extravasate and infiltrate the mucosa of the inflamed intestine, thus interacting with stromal cells, such as myofibroblasts (Sacco et al., 2019). This contributes to developing a chronic inflammatory state of the colorectum. Thus, in an animal model of intestinal colitis, the specific deletion of COX-1 in megakaryocytes/platelets, associated with the inhibition of platelet TXA_2_, promotes the resolution of chronic inflammation and ameliorates the colitis symptoms and fibrosis (Sacco et al., 2019) (Figure 2). The results of a recent study showed that tumor-platelet infiltration (TIPs) was associated with decreased postsurgical survival in CRC patients and that the combination of TIPs and the Classification of Malignant Tumours TNM staging (T category describes the primary tumor site and size; N category describes the regional lymph node involvement; M category describes the presence of distant metastatic spread) establish a nomogram with better prognostic ability than TNM alone (Miao et al., 2022). When CRC is diagnosed early and localized, the 5-year survival rate can reach about 90%. Unfortunately, according to the American Cancer Society, only four out of 10 CRC patients are detected in the early stages (https://www.cancer.org/cancer/colon-rectal-cancer/detection-diagnosis-staging/detection.html); the 5-years survival rate drops to 10% for CRC patients with distant metastasis (Haggar and Boushey, 2009). Colonoscopy is still the gold standard for CRC screening, for both early detection and primary prevention, thanks to the possibility of performing biopsies and removing precancerous lesions. However, this procedure has several limitations: 1) it is invasive with bleeding and intestinal perforation occurring in 0.001–0.687% and 0.005–0.085%, respectively; 2) it is uncomfortable and 3) it must be managed by qualified health personnel (Kim et al., 2019). The fecal occult blood test and the fecal immunochemical tests help identify CRC patients, but their sensitivity is low; these tests can detect only 27% of advanced neoplasms and 66% of invasive cancer (Morikawa et al., 2005). These data show an urgent need to have noninvasive and highly sensitive screening tools to detect CRC at the early stage accurately.

### Aspirin in the Prevention of CRC

The first evidence of the apparent chemopreventive effect of Aspirin against CRC derives from epidemiological studies. In case-control and cohort studies, daily use of Aspirin, with a regular drug consumption for at least a decade, has been associated with a reduction of about 50% in the incidence and mortality of CRC (Giovannucci et al., 1995; Thun et al., 2002; Cuzick et al., 2009).

The meta-analyses of CV RCTs with Aspirin versus placebo have shown that the drug, when chronically administered for at least 5 years, reduces the risk of cancer at the gastrointestinal (GI) tract (i.e., esophagus, stomach, and colon) by ∼20% (Rothwell et al., 2010; Rothwell et al., 2011). However, protection against other cancers, such as breast, lung, and prostate, has been detected with a lower reduction in risk (Rothwell et al., 2011). Death due to CRC on long-term follow-up after RCTs of Aspirin versus placebo showed that the protective effect was saturable at low doses (Rothwell et al., 2010). Within the limitations of post hoc analyses of cancer events that were not pre-specified endpoints, the results of the Thrombosis Prevention Trial (TPT) (Meade, 1998) are of interest since the anticancer effect of Aspirin was apparent in men at high CV risk treated with a 75-mg controlled-release aspirin formulation associated with tiny concentrations measured in the systemic circulation. This Aspirin formulation was developed to maximize cumulative inhibition of platelet COX-1 in the pre-hepatic circulation and minimize inhibition of COX-2 in the systemic compartment (Charman et al., 1993). Moreover, a reduced risk for CRC (a pre-specified secondary endpoint) was detected in the long-term observational follow-up of the Women’s Health Study where Aspirin was administered in alternate-day 100-mg Aspirin versus placebo (Cook et al., 2005). Altogether these findings suggest that the chemopreventive effect of Aspirin has features comparable to its antiplatelet effect, i.e., a long-lasting duration and, most importantly, its saturability at low doses (Patrignani et al., 1982; Patrignani et al., 2014).

In individuals with sporadic colorectal adenomas, placebo-controlled RCTs have shown a significant risk reduction of reoccurrence after treatment with Aspirin (81–325 mg daily) (Baron et al., 2003; Benamouzig et al., 2003; Sandler et al., 2003; Benamouzig et al., 2012). However, a comparable benefit was detected in the placebo-controlled RCTs with the selective COX-2 inhibitors celecoxib and rofecoxib in individuals with a history of colorectal adenomas (Bresalier et al., 2005; Bertagnolli et al., 2006). The comparable benefit of low-dose Aspirin (which targets platelet COX-1) and coxibs (which target COX-2 in extraplatelet cellular sources) has led to hypothesize that platelets contribute to adenoma development via the induction of COX-2 expression in stromal cells of colorectal mucosa and then in epithelial cells (Patrono et al., 2001; Patrignani and Patrono, 2016; Dovizio et al., 2017; Patrignani and Patrono, 2018).

Familial adenomatous polyposis (FAP) represents an accelerated clinical manifestation of the adenoma to carcinoma sequence that characterizes the development of most CRCs; the studies in FAP patients contribute to enhancing knowledge in the interaction of genetic and molecular events occurring in the development of sporadic colorectal neoplasia (Fearon and Vogelstein, 1990). The chemopreventive effect of Aspirin in FAP patients is not completely clarified yet. Colorectal Adenoma/Carcinoma Prevention Program 1 (CAPP1) is a multicenter, randomized, placebo-controlled trial performed in FAP patients (10–21 years old of both sexes) treated with 600 mg/day (two tablets/day) and/or resistant starch 30 g/d (30 g as two sachets/day) (Burn et al., 2011). The duration of intervention was from one to a potential maximum of 12 years, with a scheduled annual clinical examination including endoscopy. Among 133 evaluable patients, Aspirin treatment resulted in a nonsignificant reduction in polyp number (RR = 0.77; 95% CI, 0.54–1.10) compared with nonaspirin, and a significant decrease in the diameter of the largest polyps (principal secondary endpoint) among patients treated with Aspirin for more than 1 year. No serious adverse effects were recorded.

Recently, Ishikawa et al. (2021) performed a double-blind, placebo-controlled, multicenter trial in 104 patients (age: 16–70 years) with FAP for the effects of low-dose Aspirin (100 mg/day) and mesalazine (2 g/day) on the recurrence of colorectal polyps (J-FAPP Study IV). The primary endpoint was the incidence of colorectal polyps of at least 5 mm; treatment continued until 1 week before an 8-month colonoscopy. Polyp recurrence was significantly reduced in patients who received Aspirin versus mesalazine [OR values: 0.37 (95% CI, 0.16–0.86) for Aspirin and 0.87 (95% CI, 0.38–2) for mesalazine)]. The most common adverse events recorded were low-grade upper GI symptoms. This trial has some limitations (Lynch, 2021), including the fact that the emergence of adenomas greater than 5.0 mm is not an accurate measure of clinical benefit. Like all other chemoprevention trials for FAP, achieving reproducible endoscopic examinations from baseline to post-intervention is a challenge; thus, advanced endoscopic image capture of all the available adenoma evaluated by independent audit should be used in further trials. In J-FAPP Study IV, the average age of patients at entry was over 30 years suggesting that many patients had attenuated FAP.

**FIGURE 3:**
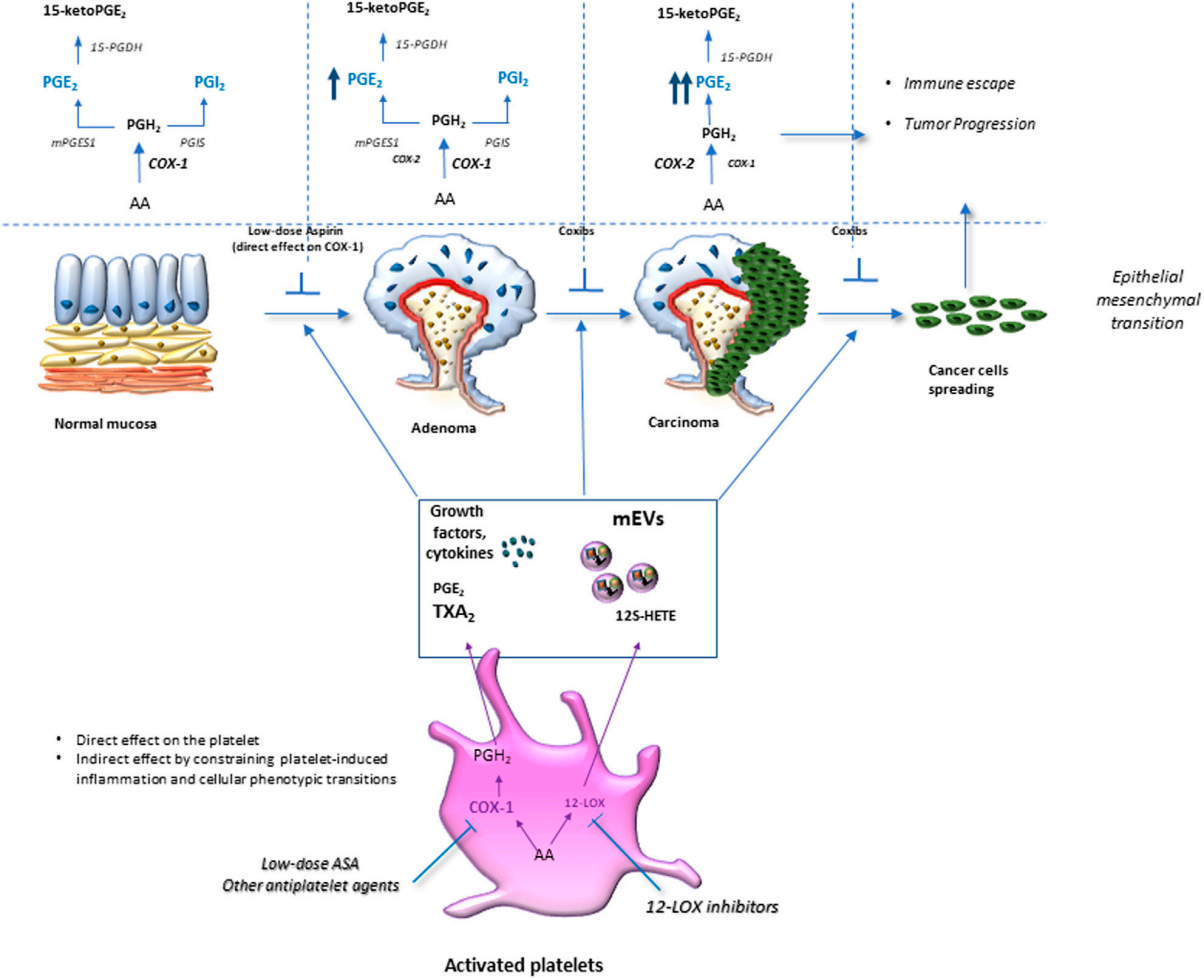
Platelet activation in response to intestinal damage is crucial in chronic inflammation/fibrosis. Platelet activation in response to intestinal epithelial damage contributes to acute inflammation by promoting leukocyte recruitment to restore normal tissue function. However, exaggerated platelet activation is associated with an elevated release of thromboxane (TX) A_2_ and prostaglandin (PG)E_2_, growth factors, angiogenic factors, cytokines, and chemokines, as well as medium-sized extracellular vesicles (mEVs) rich in genetic material (mRNAs and microRNAs). These factors activate stromal cells (such as myofibroblasts and immune cells), thus increasing the production and release of growth factors and inflammatory mediators, including PGE_2_, due to cyclooxygenase (COX)-2 induction. Platelet-derived TXA_2_ induces phenotypic and functional changes in myofibroblasts, such as the reduction of α-Alpha Smooth Muscle Actin (SMA) and the increase of vimentin fibronectin RhoA expression; these events lead to an enhanced capacity to proliferate and migrate, thus, contributing to chronic intestinal inflammation and fibrosis. The inhibition of platelet COX-1 activity (by low-dose Aspirin) or blocking the TXA_2_ receptor (TP) can mitigate chronic intestinal inflammation and fibrosis.

Dovizio et al. (2012) have reported that enhanced systemic biosynthesis of TXA_2_ occurs *in vivo* in FAP, possibly from activated platelets; in fact, it was not affected by celecoxib. Activated platelets can induce several signaling pathways, including those related to TXA_2_, in stromal and tumor cells, thus contributing to inflammatory and carcinogenic responses mediated, at least in part, by enhanced biosynthesis of COX-2-dependent PGE_2_ (Patrignani and Patrono, 2016; Patrignani and Patrono, 2018) (Figure 3). Thus, low-dose Aspirin can mitigate these effects indirectly by inhibiting platelet function. It is noteworthy to consider that high doses of Aspirin used in the CAPP1 trial could have reduced the chemopreventive effect of Aspirin due to the coincident depression of vascular PGI_2_, which exerts anticarcinogenic effects (Dovizio et al., 2012).

In Lynch Syndrome (LS) carriers, formerly known as hereditary nonpolyposis colon cancer, which results from pathogenic variants in one of the DNA mismatch repair genes (Li et al., 2021), Aspirin 600 mg/day (2 tablets/day) for 2 years resulted in 60% reduction in the incidence of CRC- and other LS-associated tumors. The protection persisted for over a decade but did not become apparent until about 5 years from the beginning. In this relatively young group of individuals, serious adverse events did not differ significantly in the Aspirin group versus the placebo group (Burn et al., 2020). These positive results support the use of Aspirin as a cancer prevention measure in individuals with LS. However, the optimal dose has still not been determined and is the objective of the ongoing CAPP3 study that compares daily Aspirin at 600, 300, and 100 mg for cancer prevention versus adverse events (http://www.capp3.org).

Overall, numerous lines of evidence support the role of the antiplatelet agent low-dose Aspirin in preventing atherothrombosis and CRC. However, its use cannot be recommended for the primary prevention of these diseases due to the possible enhanced risk of hemorrhage via inhibition of TXA_2_-dependent platelet function, an important component of primary hemostasis. The bleeding risk is smaller in young people and substantially higher in elderly individuals and those with a history of ulcer bleeding. The publication in 2018 of three placebo-controlled RCTs, i.e., ARRIVE, ASCEND, and ASPREE (ASCEND Study Collaborative Group et al., 2018; Gaziano et al., 2018; McNeil et al., 2018), in three populations at increased risk of myocardial infarction or ischaemic stroke in the absence of established CV disease show that low-dose Aspirin conferred little or no cardiovascular benefit and a small risk for major hemorrhage. In October 2021, United States Preventive ServicesTask Force (USPSTF) stated that “the decision to initiate low-dose aspirin use for the primary prevention of CV disease in adults ages 40–59 years who have a 10% or greater 10-year CV risk should be an individual one. Persons who are not at increased risk for bleeding and are willing to take low-dose Aspirin daily are more likely to benefit.” Aspirin use is best reserved for individuals with specific molecularly driven cancer risks, such as those with LS, with a low risk of bleeding due to young age.

These findings convincingly show that it is necessary to develop novel antiplatelet agents with an improved safety profile (Majithia and Bhatt, 2019) and novel technologies involved in transporting a pharmaceutical compound to its target site to achieve a desired therapeutic outcome, thus reducing systemic off-target effects (targeted drug delivery systems) (Dovizio et al., 2020). Moreover, an important field of research is the development of biomarkers predictive for the response to a drug on an individual basis (precision medicine). The ongoing ADD-Aspirin trial will contribute to realizing this objective (Coyle et al., 2016). The trial includes four phase III RCTs evaluating the effect of two doses of daily Aspirin, i.e., 100 and 300 mg/day, on recurrence and survival after radical cancer therapy in patients with four non-metastatic common solid tumors (colorectal, gastroesophageal, breast, and prostate cancer). In addition to the clinical outcomes, samples are collected to identify mechanistic biomarkers of aspirin responses.

### Experimental Evidence on the Role of Platelets in CRC

Platelets are rapidly activated to repair tissue injury/dysfunction (Gawatz et al., 2005; Dovizio et al., 2017; Tourdot and Holinstat, 2017) (Figure 2). Several functions of platelets are involved: the adhesion to extracellular matrix (ECM) proteins exposed by the tissue damage (Bergmeier and Hynes, 2012), the aggregation leading to the amplification of platelet activation (Davì and Patrono, 2007), the release of many factors such as the eicosanoids (TXA_2_ and PGE_2_ and 12S-HETE), proteins (growth and angiogenic factors) and Extracellular visicles (EVs) (Dovizio et al., 2020). EVs are of various sizes and play an important role in the delivery of platelet cargo (including proteins and transcripts, including microRNAs) to other cell types even far from the site of the tissue damage (Burnouf et al., 2014; Dovizio et al., 2020; Dovizio et al., 2018; Puhm et al., 2021). The crosstalk with EVs contributes to chronic inflammation and fibrosis by activating immune cells, fibroblasts, and endothelial cells (Figure 2). Chronic inflammation contributes to several diseases, including atherothrombosis (Soehnlein and Libby, 2021) and cancer (Hanahan and Weinberg, 2011). Lifestyle habits, such as low physical activity, excessive consumption of red, processed meats, alcohol, low dietary fiber content, and smoking can alter intestinal homeostasis, which requires the appropriate integration of various cell signaling pathways and balanced crosstalk between different cell types composing the organ (Grazioso et al., 2019). Dysregulation of this balance can impair the mucosal barrier allowing the gut bacteria to invade the mucosa inducing platelet activation and an excessive immune response (Figure 2). In an animal model of colitis, the inhibition of platelet function by selective deletion of COX-1 in platelets (which recapitulated the human pharmacodynamics of low-dose Aspirin, i.e., suppression of platelet TXA_2_ production associated with substantial sparing of the systemic prostanoid biosynthesis) ameliorates colitis symptoms, chronic intestinal inflammation, and fibrosis (Sacco et al., 2019) (Figure 2). These findings suggest that platelet activation is an upstream event in the development of chronic intestinal inflammation. Platelet-derived TXA_2_ enhances the ability of myofibroblasts to proliferate and migrate *in vitro*, and these effects were prevented by platelet COX-1 inhibition (Aspirin) or antagonism of the TXA_2_ receptor (Sacco et al., 2019). Interestingly, platelet-derived TXA_2_ enhanced the expression of mesenchymal markers, such as vimentin and fibronectin, and these effects were abrogated by incubating the myofibroblasts with platelets preexposed to Aspirin (Sacco et al., 2019) (Figure 2).

Platelets can also promote a mesenchymal phenotype in cancer cells *in vitro* (Labelle et al., 2011; Guillem-Llobat et al., 2016); mesenchymal-like cancer cells are characterized by the enhanced capacity of cell mobility *in vitro* and a prothrombotic and prometastatic potential when injected into mice (Guillem-Llobat et al., 2016). In cocultures of platelets and colorectal HT29 cancer cells, platelet-derived PGE_2_ upregulated TWIST1 via the activation of the EP4 receptor (one of the four PGE_2_ receptors) (Guillem-Llobat et al., 2016). TWIST1 encodes a basic helix-loop-helix transcription factor that contributes to EMT, a key process in the metastases formation of cancer (Yang et al., 2004; Kalluri and Weinberg, 2009). This effect was associated with the downregulation of E-cadherin, a typical marker of the epithelial phenotype (van Roy and Berx, 2008), and the upregulation of Rac1 (a small G-protein of the Rho family) involved in modulation of migration (Yang et al., 2012). Different antiplatelet agents prevented these changes, Aspirin (an inhibitor of COX-1), DG-041 (an antagonist of the PGE_2_ EP3 receptor subtype), and Ticagrelor (a P2Y12 receptor antagonist), which all reduced the release of PGE_2_ from platelets, thus preventing the activation of EP4 on HT29 cells by platelet-derived PGE_2_ (Guillem-Llobat et al., 2016). Therefore, platelets prime colon cancer cells for metastasis, and antiplatelet agents can restrain it. Contursi et al. (2021) have recently found that platelets can promote EMT in cancer cells via the transfer of platelet-type 12-lipoxygenase (LOX) contained in platelet-derived medium-sized EVs (mEVs). The cancer cells now generate 12S-HETE, considered a key modulator of cancer metastasis (Dilly et al., 2013). Interestingly, 12-HETE was mainly esterified in plasmalogen phospholipids. Modifying cancer cell phospholipids by 12S-HETE may functionally impact cancer cell biology and represent a novel target for anticancer agent development. Selective inhibitors of 12-LOX, which are in clinical development, such as ML-355 (Tourdot and Holinstat, 2017), and macropinocytosis inhibitors (Lin et al., 2018), which prevent the internalization of mEVs (Contursi et al., 2021), are potential anticancer tools.

### Role of Intestinal COX-Isozymes in CRC

In the course of inflammation, many protumorigenic signalings are activated, including the induction of COX-2 and aberrant generation of PGE_2_ (Wang and DuBois, 2018). However, in the early events of intestinal tumorigenesis, the suppression of the expression of the enzyme 15-prostaglandin dehydrogenase (15-PGDH), a prostaglandin-degrading enzyme, allows enhanced biosynthesis of PGE_2_ via COX-1, before the overexpression of COX-2 (Myung et al., 2006) (Figure 3).

PGE_2_ can phosphorylate ribosomal protein S6 (p-S6) via protein kinase A (PKA) (Moore et al., 2009). S6 regulates the 40S ribosome biogenesis transcriptional program, and its phosphorylated form is related to cell growth and tumor progression (Ruvinsky et al., 2005). Patrignani et al. (2017) have shown that the human rectal mucosal levels of p-S6/S6 significantly correlated with PGE_2_. In individuals undergoing CRC screening, low-dose Aspirin reduced the levels of p-S6/S6 in apparently healthy colorectal mucosa in association with the acetylation of COX-1 at serine 529 and an approximately 50% reduction of PGE_2_ biosynthesis (Patrignani et al., 2017).

During intestinal tumor progression, aberrant generation of PGE_2_ occurs for the coordinated overexpression of COX-2 and microsomal PGE_2_ synthase-1 (mPGES-1) (Sasaki et al., 2012). PGE_2_ activates target cells by binding to four subtypes of G protein-coupled receptors (EP1, EP2, EP3, and EP4) expressed on the plasma membrane and/or nuclear envelope (Ricciotti and FitzGerald, 2011). This prostanoid promotes intestinal tumorigenesis via EP2 and EP4, but not EP3 (Wang and DuBois, 2018). The role of EP1 in CRC remains unclear. PGE_2_ promotes tumor epithelial cell proliferation, survival, migration/invasion, and epigenetic changes and contributes to the metastatic spread by inhibiting immunosurveillance and inducing angiogenesis (Wang and DuBois, 2018 Zelenay et al., 2015) (Figure 3). Thus, coxibs’ inhibition of COX-2-dependent PGE_2_ reduces the risk of sporadic colorectal adenoma recurrence (Bresalier et al., 2005; Bertagnolli et al., 2006). However, as reported above, coxibs’ use is hampered by their interference with CV homeostasis for the coincident inhibition of vascular COX-2-dependent PGI_2_ biosynthesis, resulting in enhanced risk of atherothrombosis (Grosser et al., 2006; Patrignani and Patrono, 2015).

## Hepatocellular Carcinoma

### Inflammation, Platelets, and HCC

Hepatocellular carcinoma (HCC) is the most common form of liver cancer and accounts for 75–85% of cases (Bray et al., 2018). Hepatitis B virus (HBV) and hepatitis C virus (HCV) infections are responsible for about 50% of liver cancer mortality followed by alcohol consumption (∼30%) and by other causes (∼15%) (Akinyemiju et al., 2017).

HBV is a non-cytopathic, hepatotropic DNA virus that integrates into the host genome (Wang et al., 1990). A direct oncogenic activity has been described for the truncated and mutated HBV proteins, mainly HBx or preS2/S. These proteins contribute to hepatocarcinogenesis by affecting different signal transduction pathways, including inflammatory responses (Hai et al., 2014). HBx has been shown to induce COX-2 expression and PGE_2_ biosynthesis when transfected in Hep3B hepatocellular carcinoma cells, characterized by low levels of COX-2 (Cheng et al., 2004). More recently, it has been reported that HBV activates the expression of COX-2 and PGE_2_ biosynthesis in HepG2 cells and Huh7 human hepatoma cells co-transfected with plasmid pHBV-1.2 (to produce mature HBV virions) and with the reporter plasmids pGL3-COX-2-Luc (Yu et al., 2011).

HCV is an RNA virus. It is a hepatotropic, principally non-cytopathic virus with a high mutation rate; it is one of the most important risk factors for HCC. The viral protein NS5A has been reported to induce COX-2 promoter activity in a concentration-dependent manner in Huh7 human hepatoma cells (Chen et al., 2019).

Although infection by HBV and HCV remain the main risk factors for HCC development, nonalcoholic fatty liver disease (NAFLD), a pathological liver condition associated with obesity, insulin resistance, or metabolic syndrome, is becoming a more frequent risk factor in the western countries characterized by a high socio-demographic index (Pennisi et al., 2019). NAFLD includes a spectrum of pathological conditions ranging from simple steatosis (NAFL) to steatohepatitis (NASH), which can lead to cirrhosis in approximately 9–20% of patients during 5–10 years and eventually in HCC (Bessone et al., 2019). In this scenario, it is worth mentioning that NASH and older age are expected to cause the most marked increase of primary liver cancer cases in 2030 (Liu et al., 2021).

NAFLD progression results from a complex integrated combination of different not yet fully elucidated pathogenic molecular events (Tilg and Moschen, 2010; Yilmaz, 2012). Inflammation in NASH is driven by liver cells and non-hepatic tissues, such as adipose tissue, which produce several pro-inflammatory cytokines (e.g., TGF-α, IL-1β, IL-6, IL-8) and chemokines (e.g., MCP-1). Inflammation has been associated with the development of liver fibrosis found in a high percentage of NASH patients (from 37 up to 84%) and is a negative prognostic factor. As for viral hepatitis, in NASH, resident hematopoietic stem cells (HSCs) are the primary effector cells responsible for ECM protein production, mainly of type I and type II collagen. Also, extrahepatic cells may play a role in modulating HSC-mediated pro-fibrotic effects, including sinusoidal endothelial cells and platelets. Indeed, platelet-derived growth factor (PDGF) is one of the most potent factors reported to: 1) stimulate HSC proliferation, differentiation, and migration; 2) promote HSC-mediated collagen production and deposition; 3) favor HSC transformation into myofibroblasts (Kocabayoglu et al., 2015). A significant increase in platelet number and aggregates was found in livers of C57Bl/6 mice fed with a choline-deficient, high-fat diet compared with standard chow diet-fed animals (Malehmir et al., 2019). A similar result was described in NASH patients who displayed an increased number of platelets in the liver samples compared to healthy controls (Malehmir et al., 2019).

In the chronically injured liver, unresolved inflammation plays a predominant role in liver fibrosis by activating HSCs, which acquire the ability to migrate and accumulate at sites of tissue repair (Bataller and Brenner, 2005). Liver fibrosis is the excessive accumulation of extracellular matrix proteins, including collagen, occurring in most types of chronic liver disease. Advanced liver fibrosis results in cirrhosis, liver failure, and portal hypertension and often requires liver transplantation. Cirrhosis represents the higher risk factor for HCC (Marrero et al., 2018). In cirrhotic patients, HCC is the leading cause of death, with a reported annual incidence in the range of 1–6% (Trinchet et al., 2015).

Developing reliable noninvasive biomarkers for early detection of liver fibrosis and its eventual progression towards cirrhosis is an important goal to realize in this setting. Most importantly, the efficacy of antifibrotic drugs known to attenuate experimental liver fibrosis should be tested in humans (Bataller and Brenner, 2005).

Most patients with HCC are diagnosed at a late stage of the disease with a median survival of less than 1 year, whereas when detected at early-stage, patients can reach a survival rate of 70%, thanks to liver resection or transplant (Singal et al., 2014). During the last years, the incidence of NAFLD, the primary cause of chronic liver disease, has increased in association with the rise in the prevalence of other metabolic disorders and has reached a global prevalence of 25% (Younossi et al., 2016). About 10–20% of NAFLD patients progress towards NASH, leading to cirrhosis and liver-related mortality. HCC increases up to 5.29 per 1,000 person-years after NAFLD-to-NASH transition (Younossi et al., 2016).

Currently, liver biopsy represents the only accepted method to differentiate NASH from simple steatosis. However, liver biopsy can be considered neither an acceptable first-line investigation nor an acceptable method to monitor disease progression and/or drug response. It is associated with several risks, including potentially fatal bleeding and tumor seeding. On the other hand, proposed noninvasive steatosis biomarkers such as fatty liver index have shown limited clinical utility since they cannot quantify the presence of steatosis with the same accuracy as histological analysis (Haggar and Boushey, 2009).

A better understanding of the pathogenesis of NAFLD and the mechanisms underlying NALFD-to-NASH transition and related fibrosis is crucial for developing reliable, noninvasive biomarkers that allow early diagnosis and prognosis and monitor the progression of this complex pathological condition towards HCC.

### Aspirin in the Prevention of HCC

Several epidemiological studies performed in the general population or selected patients have shown that Aspirin (at different doses) was associated with a significantly lower risk of HCC and liver-related mortality than no Aspirin use (Sahasrabuddhe et al., 2012; Petrick et al., 2015; Hwang et al., 2018; Simon et al., 2018; Lee et al., 2019; Simon et al., 2020).

Pooled analysis of two prospective United States cohort studies (the Nurses’ Health Study and thistle Health Professionals Follow-up Study) (Colditz et al., 1986; Chasan-Taber et al., 1996) reported that regular Aspirin use [defined as consumption of two or more standard-dose (325-mg) Aspirin tablets per week] was associated with reduced HCC risk (adjusted HR, 0.51; 95% CI, 0.34–0.77) (Simon et al., 2018). This reduction of risk was dose-dependent [> 1.5 to 5 tablets per week (*p* = 0.006)] and time-dependent (becoming apparent when Aspirin was given for five or more years, *p* = 0.03). The protective effect of Aspirin was reported for individuals with or without cirrhosis. More recently, Simon et al. (2020) examined the impact of low-dose Aspirin (75 or 160 mg/day) on two primary outcomes, i.e., the incident HCC and liver-related mortality, in the Cancer and Cause of Death registries in Swedish adults with confirmed chronic hepatitis B or hepatitis C infection. A reduction of the estimated cumulative incidence of HCC was 31% at 7.9 years of follow-up versus nonusers. Liver-related mortality was also reduced by 27% in aspirin users versus nonusers. Lower risk of HCC was detected after 3–5 years of use. Aspirin benefits were not associated with a higher incidence of gastrointestinal bleeding (Simon et al., 2020).

In a retrospective Taiwan nationwide cohort study, HBV patients taking Aspirin (mainly 100 mg/day) showed a significant (*p* < 0.001) lower 5-year cumulative incidence of HCC versus the untreated group (*p* < 0.001) (Lee et al., 2019). The multivariable regression analysis showed that low-dose Aspirin represented an independent factor associated with a risk reduction of HCC development. However, older age, male sex, and liver cirrhosis were associated with a higher HCC risk. The use of antiviral medicines, such as nucleos(t)ide analog (NA) or statins, was associated with a lower HCC risk. The beneficial chemopreventive effect of Aspirin was evident after 2 years of aspirin therapy. In addition, the multivariable stratified analysis for aspirin therapy showed that statistical significance was not reached in several patient subgroups, including those with underlying cirrhosis, NA, and statin users. Some systematic reviews of published observational studies were recently performed to evaluate the association between the use of Aspirin and the incidence of HCC (Memel et al., 2020; Wang et al., 2020). It was confirmed that aspirin use significantly reduces the risk of HCC versus nonusers. The association between aspirin use and HCC risk was modestly or nonattenuated in populations with liver disease.

While several preclinical data suggest that Aspirin may protect against liver fibrosis (Yoshida et al., 2014; Li et al., 2017), clinical evidence of aspirin efficacy on fibrosis in patients with NAFLD remains scarce. Two cross-sectional studies were performed in NAFLD treated with Aspirin (Jiang et al., 2016; Devaki and McCullough, 2017). In Devaki’s study, Aspirin administration was inversely associated with steatosis, defined by abdominal ultrasound. In Jiang’s study, Aspirin treatment was associated with lower serum markers of hepatic fibrosis. However, these studies presented important limitations, such as the cross-sectional design and lack of hepatic histology at the severity stage of NAFLD. Simon et al. (2019) performed a prospective cohort study of 361 adults with biopsy-confirmed NAFLD. They demonstrated that daily Aspirin administration was associated with less severe histologic features of NAFLD and NASH and a reduced risk for the progression to advanced fibrosis. These results were consistent in women and men, and the inverse relationship was duration-dependent. Similar associations were not found with nonaspirin NSAIDs users, i.e., anyone reporting nonaspirin NSAID use at least twice weekly or who received an NSAID prescription (i.e., ibuprofen, naproxen, ketoprofen, diclofenac, indomethacin) at least twice weekly.

In summary, the majority of the evidence linking aspirin use in the protection against NAFLD, NASH, or HCC arises from observational studies (Ricciotti et al., 2021). These studies often miss some critical information necessary to make mechanistic interpretations on aspirin effects, such as the dose and the duration of aspirin therapy, the safety, alcohol consumption, smoking habit, fibrosis stage, cancer stage, use of other potential chemopreventive drugs.

### Experimental Evidence on the Role of Platelets in HCC

**FIGURE 4:**
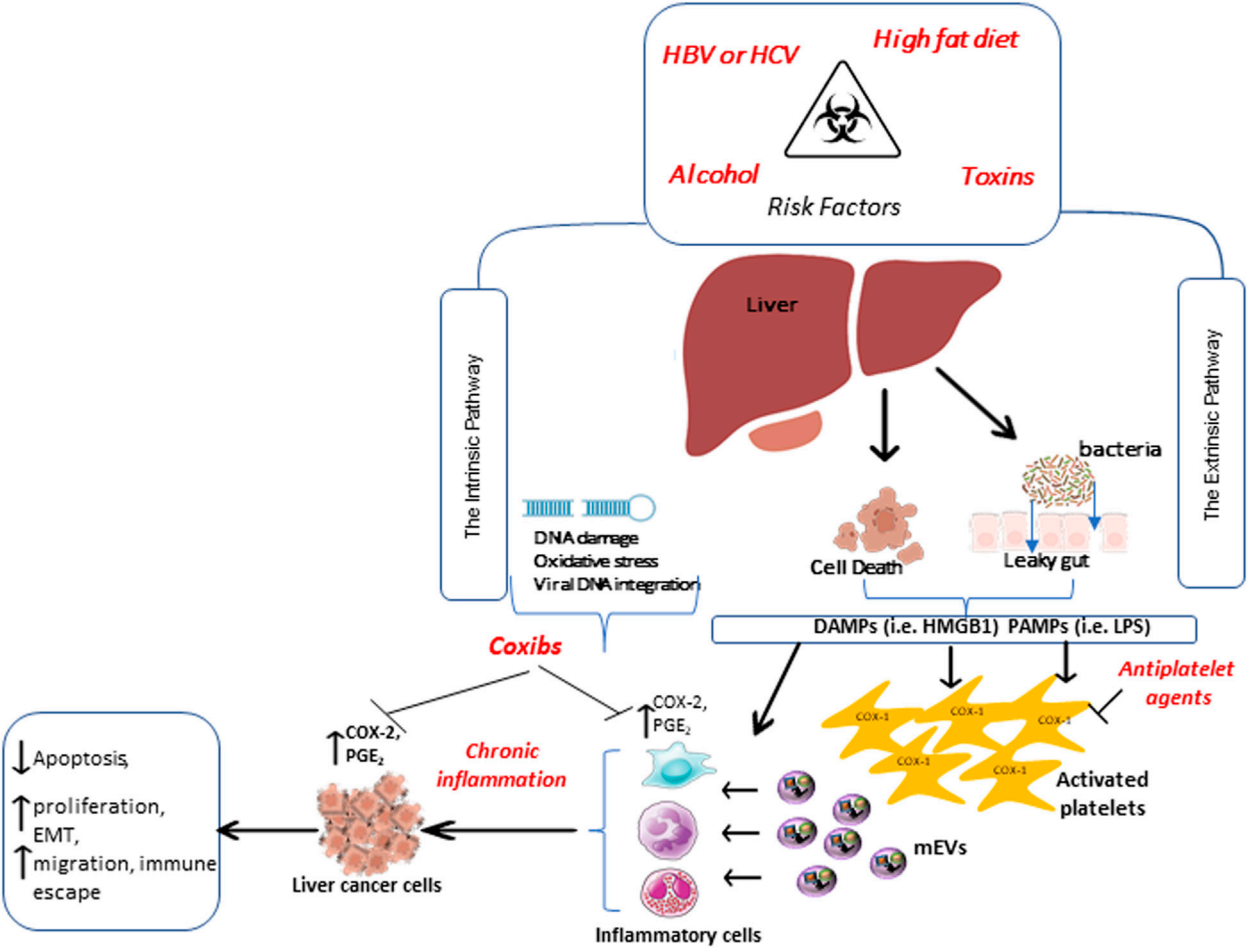
Extrinsic and intrinsic pathways promote chronic inflammation involved in developing hepatocellular carcinoma. Two distinct pathways are involved in chronic inflammation during hepatocarcinogenesis. The extrinsic pathway is triggered by exogenous factors, such as pathogen-associated molecular patterns (PAMPs) or damage-associated molecular patterns (DAMPs) from dying cells. These, in turn, are recognized by specific receptors expressed in inflammatory cells and platelets. PAMPs and DAMPs activate platelets, which release medium-sized extracellular vesicles (mEVs) able to exit the vasculature, reach tissues, and sustain inflammation, thus increasing cancer risk. The intrinsic pathway, induced by alteration in cancer-associated genetic factors, activates the expression of inflammation-related programs, thus contributing to the generation and perpetuation of an inflammatory milieu. All these events further contribute to the overexpression of COX-2 and enhanced PGE_2_ biosynthesis, promoting the transformation of normal epithelial cells to cancer. Antiplatelet drugs, such as low-dose Aspirin, can affect the early step of tumor development by inhibiting platelet function. In contrast, selective COX-2 inhibitors (coxibs) may exert an antitumor effect by inhibiting the synthesis of PGE_2_ in leukocytes and tumor cells.

HCC represents a classic paradigm of inflammation-linked cancer, as more than 90% of HCCs arise in the context of hepatic injury and inflammation (El-Serag and Rudolph, 2007). Two distinct pathways can develop chronic inflammation during hepatocarcinogenesis (Yu et al., 2018) (Figure 4). The extrinsic pathway is driven by exogenous factors (e.g., the PAMPs from pathogens or DAMPs from dead cells), triggering a persistent inflammatory response by engaging the receptors expressed in the inflammatory cells and establishing an inflammatory condition that increases cancer risk. Platelets possess receptors that respond to PAMPs and DAMPs; their activation triggers hemostatic and inflammatory responses against bacterial and viral infections (Jenne, 2014). Activated platelets produce mEVs during bacterial (Ogura et al., 2001) and viral infection (Mayne et al., 2012) that can exit the vasculature and enter tissues to activate immune and stromal cells to drive the inflammatory response further. For example, platelet mEVs enhance the expression of cell adhesion molecules such as leukocyte αMβ2 (Mac-1, CD11b/CD18) for monocyte adhesion (Barry et al., 1998). Levi et al. (2017) reported that platelet mEVs were significantly elevated in the blood of patients with HCC. Importantly, platelet-derived mEVs can induce the expression of marker genes of EMT in cancer cells and endothelial mesenchymal transition (EndMT) in human microvascular endothelial cells (Grande et al., 2019).

Altogether this knowledge sustains a central role of platelet activation in HCC. Numerous studies have been performed in mouse models to verify the impact of antiplatelet agents. In chronic HBV immune-mediated HCC, platelet activation contributed to the accumulation of virus-specific CD8^+^ T cells and virus-nonspecific inflammatory cells in the liver. In this model, the administration of Aspirin and/or Clopidogrel [an antagonist of the platelet P2Y12 receptor for ADP (Ballerini et al., 2018)] reduced inflammation and immune cell infiltration (Sitia et al., 2012). The two drugs reduced liver fibrosis, HCC progression, and survival when co-administered. These effects were not associated with increased bleeding (Sitia et al., 2012). The antitumor effect of Aspirin and Clopidogrel was also confirmed in a recent study, where this combination prevented nonalcoholic steatohepatitis and subsequent HCC development in different dietary and genetic mouse models (Ribas et al., 2021). This study also found that another P2Y12 inhibitor, Ticagrelor, had a similar effect. Differently, nonaspirin NSAIDs did not affect the development of nonalcoholic steatohepatitis and HCC in this setting. The findings that the two antiplatelet agents (Aspirin and Clopidogrel) reduced liver fibrosis and HCC progression suggest that the inhibition of platelet activation was the central mechanism involved in their beneficial effects.

However, other studies performed *in vitro* and *in vivo* at supratherapeutic concentrations of Aspirin evidenced the capacity of the drug to cause extraplatelet effects (Ricciotti et al., 2021). Li et al. (2013) showed that 1 mM of Aspirin enhanced the anti-proliferative and pro-apoptotic action induced by IFN through the phosphorylation of STAT-1. In a xenograft model in nude mice, Aspirin (15 mg kg/day) reduced both tumor growth and the number of STAT1-expressing apoptotic cells present in the tumor (Li et al., 2013). Whether the dose of Aspirin administered to mice was selective for the platelets or caused systemic effects was not explored. In another study, Aspirin reduced the expression of collagen prolyl 4-hydroxylase α2 (P4HA2), the essential enzyme during collagen formation, in HCC cells (Wang et al., 2019). Also, in this study, the *in vitro* effects were studied at millimolar concentrations of Aspirin, which are supratherapeutic. In xenograft mice, Aspirin used at a high dose (75 mg/kg/day) affected P4HA2 to decrease collagen deposition, inhibiting liver tumor growth. In contrast, mice overexpressing P4HA2 had increased collagen deposition in the liver and larger tumor size than wild-type xenograft mice (Wang et al., 2019).

To summarize, the efficacy demonstrated by different antiplatelet agents convincingly supports the platelets’ hypothesis in the development of HCC. In contrast, some studies performed *in vitro* in cell cultures or *in vivo* in animal models with Aspirin used very high concentrations/doses, which are not reached even after administering anti-inflammatory doses of Aspirin. Although these studies are not appropriate to clarify the mechanism of the apparent anticancer effect of Aspirin detected in clinical studies, their results have evidenced possible new targets for developing novel therapies to prevent and treat HCC.

The other pathway contributing to developing chronic inflammation during hepatocarcinogenesis is the intrinsic one (Yu et al., 2018) induced by alterations in cancer-associated genetic factors (e.g., mutation of either oncogenes or tumor suppressor genes), which activate the expression of inflammation-related programs (Figure 4). In this scenario, the upregulation of COX-2 is noteworthy. COX-2 expression is increased in injured livers and human HCC (Shiota et al., 1999; Cheng et al., 2004) and is associated with reduced overall survival in HCC (Chen et al., 2016). *In vitro* and *in vivo*, COX-2 inhibition constrains cell growth of liver cancer cells due to the inflammation reduction and apoptosis induction (Foderà et al., 2004; Fredriksson et al., 2011; Chu et al., 2018). Thus, the coxibs could be effective in HCC prevention and progression, but the possible increased cardiovascular risk associated with their use limit this therapeutic strategy. The development of drug target therapies with anti-inflammatory agents could be an exciting approach to develop safer and more effective treatments.

## Liquid Biopsy and Drug Delivery System Development: The Contribution of Platelets

In oncology, although highly informative, the canonical analysis of tumor tissue provides a static and partial image of a pathological condition in continuous transformation, and it does not consider the evolution and time in which the disease has developed. A reliable and comprehensive characterization of tumor cell genetic profile may guide the choice of drug treatments based on the individual patient characteristics toward a more effective and safer personalized therapy approach. However, the development of noninvasive biomarkers of early disease detection is not completely realized.

In the last few years, the suitability of liquid biopsy has opened the way to novel strategies to provide valuable information about tumor biology (Pantel and Speicher, 2016). Liquid biopsy is minimally invasive allows for longitudinal monitoring of disease progression and assessment of therapy failure/resistance even before clinical or radiographic progression is evident; its use can improve cancer patients survival chances (Roschewski et al., 2016). Primary sources of liquid biopsy for biomarker detection include circulating free DNA (cfDNA) or circulating tumor DNA (ctDNA), circulating tumor cells (CTCs), extracellular vesicles, and recently platelets (Aravanis et al., 2017; Babayan and Pantel, 2018; Best et al., 2018; Heitzer et al., 2019).

Best et al. (2015) explored the possible analysis of TEPs to acquire information about the clinical condition of individuals. TEP RNA assessment can serve as a biomarker trove to detect and classify cancer via self-learning support vector machine (SVM)-based algorithms (Best et al., 2015); this highly multiplexed biomarker signature detection platform is called thromboSeq. They also investigated the potential and origin of spliced RNA profiles from TEPs for the noninvasive detection of early- and late-stage non-small-cell lung cancer (NSCLC) (Best et al., 2017). Biomarker panels to discriminate patients with NSCLC from healthy individuals and patients with various noncancerous inflammatory conditions were identified using particle-swarm optimization (PSO) driven algorithms (Best et al., 2017). The biological mechanisms responsible for TEP RNA signatures remain to be identified. However, it can be involved: 1) altered megakaryocytic RNA expression, 2) enrichment of reticulated platelets in patients with NSCLC, 3) induction of splicing, possibly partially mediated by RNA-binding protein (RBP) activity and upstream regulatory kinases such as CLK, 4) sequestration of RNAs and 5) alternative splicing events (Best et al., 2017).

A study by Clancy et al. proposed the existence of an RNA profile that discriminates platelets over their half-life (Clancy et al., 2017), distinguishing old platelets from the young ones based on their size. Notably, cancer patients show larger platelets than healthy subjects, and these platelets exhibit an abundance of genes that regulate homeostasis. These changes could explain the increased thrombotic potential in cancer (Clancy et al., 2017).

Platelets are active players in tumor progression (Patrignani and Patrono, 2016; Patrignani and Patrono, 2018). Among the numerous mechanisms described, the ability of platelets to extravasate into the tumor microenvironment is notable (Morrell et al., 2014). Once there, platelets interact with tumor cells and promote the metastatic process by many mechanisms, including EMT and COX-2 induction (Kalluri and Weinberg, 2009; Dovizio et al., 2013; Guillem-Llobat et al., 2016).

**FIGURE 5:**
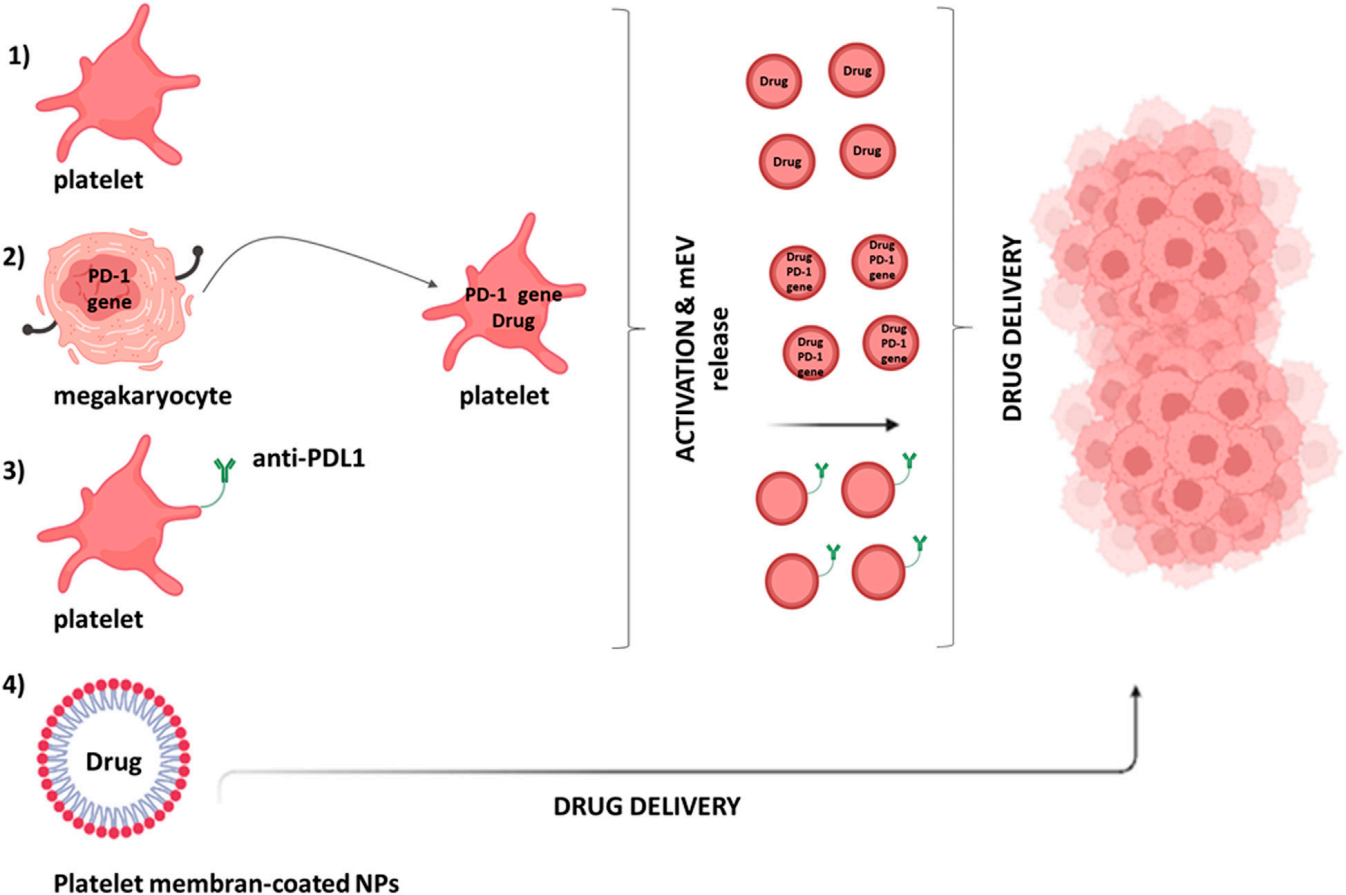
Platelet’s contribution in drug delivery systems. Different approaches can be used to deliver drugs to the tumor microenvironment by platelets: (1) platelets can be loaded with the anticancer drug via the open canalicular system; (2) genetically modified megakaryocytes (MKs) can give rise to mature platelets expressing programmed death-1 (PD-1) [involved in the immune checkpoint PD-1/programmed death-ligand 1 (PDL-1)] that can also be loaded with the anticancer drug; (3) monoclonal antibodies against PDL-1 (aPDL1) can be covalently bound to resting platelets; (4) melanin nanoparticles (MNPs) can be coated with platelet membranes.

Due to their propensity to interact with cancer cells, platelets have aroused great interest in developing cell-based strategies for drug delivery (Dovizio et al., 2020). Platelet-based approaches to drug delivery depend on: 1) low invasiveness during platelet collection from patients; 2) possibility of *in vitro* manipulation and reintroduction in the patient; 3) relatively long half-life (8–10 days), which could improve the pharmacokinetics of some drugs (Ziegler et al., 2017). Platelets can act as carriers of potent anticancer drugs such as Doxorubicin, which inhibits topoisomerase II (Sarkar et al., 2013) (Figure 5). The doxorubicin-loaded platelet delivery system effectively treated lymphoma by reducing tumor size in a mouse model obtained after injection of Burkitt lymphoma cells (Xu et al., 2017).

The application of genetic engineering to modify platelets for drug delivery applications is limited because platelets are anucleated and fully differentiated. However, their progenitor or precursor cells can be manipulated to obtain modified platelets for well-defined applications (Sim et al., 2017). Genetically modified megakaryocytes (MKs) have been created that can give rise to mature platelets expressing programmed death-1 (PD-1) and loaded with cyclophosphamide, an inhibitor of regulatory T cells (Treg) (Scurr et al., 2018) (Figure 5). In a mouse model transplanted with B16F10 melanoma tumor cells, these platelets, expressing PD1, blocked PDL-1, thus inhibiting the activity of immunosuppressive Tregs and were able to promote the anticancer activity of CD8^+^ T lymphocytes (Scurr et al., 2018).

On the other hand, platelets have numerous binding sites on their plasma membrane and can also be non-engineered to act as drug carriers (Figure 5). One example is monoclonal antibodies against programmed death-ligand 1 (aPDL1) covalently bound to resting platelets (Wang et al., 2017) released via the formation of mEVs. The administration of platelet-bound anti-PDL1 significantly reduced tumor recurrence and increased survival in experimental metastasis models (Wang et al., 2017).

To further improve their targetability, the surface of platelets was embellished with HSCs (Hu et al., 2018). These modifications induced a lack of platelet-HSC aggregation, enhancing the immune response and inhibiting leukemia progression.

Recently, nanoparticles coated with platelet membranes have been considered for drug delivery (Figure 5). Thus, melanin nanoparticles (MNPs) and Doxorubicin (DOX) were encapsulating inside platelet-derived vesicles coupled with the RGD peptide on the surface (Jing et al., 2018). They effectively suppressed tumor growth in a model where MDA-MB-231/ADR cells (adriamycin-resistant breast cancer cells) were subcutaneously implanted in BALB/c nude mice (Jing et al., 2018). Platelet membrane-coated nanoparticles have also been used in radiotherapy to improve the treatment of solid tumors; their use in a mouse model of breast cancer leads to a significant reduction in the mass and volume of the tumor itself (Chen et al., 2019).

## Conclusion and Perspectives

Considerable experimental and clinical evidence sustains chronic inflammation as an essential cancer driver. However, developing an anti-inflammatory therapy is particularly challenging in oncology (Nathan and Ding, 2010). Potent inhibitors of inflammation can lead to the possibility of increasing the risk of infections. Coxibs’ use can be associated with an enhanced risk of thrombotic events (Grosser et al., 2006). In the CANTOS trial (Canakinumab Anti-inflammatory Thrombosis Outcomes Study) (Ridker et al., 2017), Canakinumab, an IL-1β-neutralizing antibody, significantly diminished cardiovascular event rates, and the retrospective analysis showed a marked reduction (50%) in lung cancer incidence, particularly at the high-dose. However, the use of Canakinumab as a first-, second-, or third-line treatment with chemotherapy in NSCLC did not confirm the benefit (CANOPY-1 and CANOPY-2 trials) (https://bit.ly/3l0IISp). However, subgroup analysis showed a potential anticancer effect on individuals with circulating inflammation biomarkers.

The development of noninvasive biomarkers predictive of disease susceptibility or drug response is still an unmet clinical need. A range of cancer biomarkers can be found in TEPs, but their utility warrants further prospective validation (Antunes-Ferreira et al., 2021). In particular, it remains to define and validate specific and reproducible transcriptomic profiles associated with cancer that distinguish from noncancerous processes and therapeutics (such as antiplatelet agents and anticoagulants) (Antunes-Ferreira et al., 2021).

A crucial clinical need is the development of drug delivery technologies for anti-inflammatory and chemotherapeutic agents to improve safety and efficacy by enhancing the delivery of a therapeutic to its target site, minimizing off-target accumulation, and facilitating patient compliance (Vargason et al., 2021). An interesting approach can be via platelet-mediated drug delivery systems by exploiting the platelet’s ability to interact with tumor cells and transfer their molecular cargo (Dovizio et al., 2020).

Novel disease mechanisms have been proposed in CRC and HCC, enlightening the contribution of platelets in the triggering and maintaining an unresolved state of inflammation (i.e., chronic inflammation) (Patrignani and Patrono, 2016; Dovizio et al., 2017; Patrignani and Patrono, 2018). This knowledge opens the way to novel therapeutic strategies with antiplatelet agents and compounds interfering with the platelet-derived mEV uptake and cargo delivery to recipient cells. Developing personalized therapies with low-dose Aspirin and other antiplatelet agents is necessary to improve anticancer efficacy and reduce the major bleeding associated with their use.

Future clinical studies should be performed incorporating clinical and biomarker endpoints analyzed with a systems biology approach which will allow identifying dynamic systems modeling of candidate pathways involved in the antineoplastic effect of chemotherapeutic agents. This strategy will also identify susceptibility profiles for efficacy and toxicity of drug treatments in cancer.
